# Diabetes, Hypertension and Cardiovascular Disease: Clinical Insights, Mechanisms and Pharmacotherapies

**DOI:** 10.3390/medicina60040566

**Published:** 2024-03-30

**Authors:** Ming-Jui Hung

**Affiliations:** 1 Section of Cardiovascular Imaging, Division of Cardiology, Department of Internal Medicine, Chang Gung Memorial Hospital at Keelung, Keelung 20401, Taiwan; hmj1447@cgmh.org.tw; 2 College of Medicine, Chang Gung University, Taoyuan 33302, Taiwan

Cardiovascular disease (CVD) is a serious issue demanding world attention, not only because of its role in increased mortality, but also in conjunction with the aging population and growing prevalence of other co-morbidities, such as hypertension, diabetes, etc. In 2019, CVD was responsible for 33% of all-cause deaths [1]. For many years, social and academic groups have vigorously emphasized the importance of lifestyle and behavior adjustment [2]. Approximately 19.1% and 22.2% of deaths among men and women, respectively, may be attributed to five modifiable risk factors, i.e., body mass index, current smoking, systolic blood pressure, non-high-density lipoprotein cholesterol, and diabetes mellitus. Among these modifiable risk factors, hypertension, dyslipidemia, and diabetes are considered as co-morbidities. With the advancement of medical research, new drugs and medical materials have become available for the treatment of CVD and its co-morbidities. For example, several novel therapies, such as sodium glucose co-transporter 2 inhibitors, have recently been shown to be of benefit when treating CVD in patients with type 2 diabetes, and in patients with heart failure. At the same time, these medical advances have also extended our average lifespan. For these reasons, there is a need for updated clinical studies in these fields, aiming to optimize the outcomes of patients with diabetes, hypertension, and cardiovascular diseases. No single mechanism can tell the whole story of CVD and its co-morbidities; therefore, continuing medical research is needed as the driver of medical advancement. 

This Special Issue, “Diabetes, Hypertension and Cardiovascular Disease: Clinical Insights, Mechanisms and Pharmacotherapies”, was designed as a forum for authors to share important findings from their medical research focusing on diabetes, hypertension, and other cardiovascular diseases, and for others to organize and review the essence of their recent research. The published articles cover a wide range of cardiovascular-related topics, ranging methodologically from plasma biomarkers, prognostic assessment, and big data analyses to the applications of Chinese medicine. These articles underscore some important issues that we have to face and deal with in daily practice.

For coronary artery disease, Hung et al. [3] review recent advances regarding epicardial coronary artery spasm-induced vasospastic angina, one of the very important causes of myocardial ischemia with no obstructive coronary arteries (INOCA). Local and systemic inflammation can be found in patients with vasospastic angina. Therefore, treatment strategies to decrease inflammation might be effective for these patients. However, the importance of correct diagnosis as the first step in achieving effective treatment cannot be overemphasized. In other words, it is important to identify the underlying cause of INOCA, especially in this era of primary coronary intervention, because inaccurate diagnosis can lead to insufficient treatment and poor prognosis, such as the development of heart failure [4]. 

In addition to the traditional evaluation of the association between biomarkers and coronary artery disease, Yang et al. [5] explore the use of three-dimensional anthropometric body surface scanning measurements. They show that these scanning measurements, in combination with leptin, adiponectin and interleukin-6 levels, offer the best level of discrimination to identify individuals at risk of coronary artery disease. 

Regarding peripheral artery disease, Onofrei et al. [6] evaluate the role of novel inflammatory markers in assessing the severity of peripheral artery disease. Their novel inflammatory markers include the neutrophil-to-lymphocyte ratio, platelet-to-lymphocyte ratio, and lymphocyte-to-C-reactive protein ratio. These three markers have important roles in treatment benefits and prognostic implications. 

Obesity is suggested to have paradoxical survival benefits in patients with heart failure. Alrob et al. [7] identify an inverted U-shaped relationship between body mass index and left ventricular ejection fraction in patients with heart failure and reduced ejection fraction. Therefore, they propose the existence of an obesity paradox among patients with these conditions.

Regarding heart failure, Tsai et al. [8] use a large data collection from the Chang Gung Research Database to analyze the effects of heart rate reductions on the prognosis of patients with decompensated heart failure and reduced ejection fraction. A greater reduction in heart rate after hospital discharge is associated with a better prognosis. Hence, they suggest that achieving a target heart rate reduction leads to a lower prevalence of cardiovascular death, hospitalization for heart failure, and all-cause mortality.

For hypertension, Aursulesei Onofrei et al. [9] assess the prognostic value of the subendocardial viability ratio (SEVR), i.e., the Buckberg index, in patients with hypersensitivity. They describe how SEVR is associated with age, central and peripheral systolic blood pressure, heart rate, serum fibrinogen, and serum hemoglobin. The prognostic value of SEVR in hypertension is suggested by modulating the Framingham Risk Score values and Systemic Coronary Risk Evaluation risk values.

Addressing safety concerns about the use of idarucizumab for the reversal of the direct oral anticoagulation agent, dabigatran, Dai et al. [10] retrospectively analyze a cohort study based on electronic medical records from the Chang Gung Research Database in Taiwan. They suggest that idarucizumab can safely and effectively be used to reverse the anticoagulant effect of dabigatran. This result provides real-world evidence for the application of idarucizumab.

Joo et al. [11] report on the hypoglycemic effect of modified Gangsimtang, an herbal decoction, on a patient with type 2 diabetes mellitus who refused to undergo conventional therapies. No adverse events were reported over 200 days of follow-up.

To evaluate the efficacy and safety of the Compounded Danshen Dripping Pill (CDDP) with regard to blood viscosity in patients with type 2 diabetes mellitus, Wi et al. [12] systemically searched seven databases that used CDDP to treat type 2 diabetes mellitus. Although their meta-analysis shows that CDDP reduces blood viscosity in type 2 diabetes mellitus, further high-quality, well-designed studies are needed to provide more solid evidence for its future applications.

MicroRNA is found to play important roles in the pathophysiology of diabetic kidney disease. Lee et al. [13] examine the potential applications of urinary microRNA on diabetic kidney disease from the emerging experimental and clinical evidence. While the results of using urinary microRNA as a non-invasive disease marker to predict diabetic kidney disease appear promising, further experimental standardization and clinical verification trials are warranted to promote its application practically. 

Glucagon-like peptide receptor agonists (GLP-1 RAs) are proven to reduce glucose levels in patients with type 2 diabetes; their evidence of safety and prognostic implications remain to be further evaluated in relation to different drugs in the same class. Hu and Tsai et al. [14] found that semaglutide is associated with better outcomes for heart failure and cardiovascular death in patients classed as non-diabetic obese, whereas liraglutide is associated with worse outcomes of heart failure in patients with diabetes with a reduced ejection fraction. The GLP-1 RAs could reduce macroalbuminuria but could not improve renal function. The GLP-1 RAs still provide benefits in patients with type 2 diabetes or obesity.

With the publication of this Special Issue, its readers will have an opportunity to find useful information for their future research interests, including some helpful solutions for the challenges they encounter in their own works.
